# Thermoelectric effect in an Aharonov-Bohm ring with an embedded quantum dot

**DOI:** 10.1186/1556-276X-7-157

**Published:** 2012-02-28

**Authors:** Jun Zheng, Feng Chi, Xiao-Dong Lu, Kai-Cheng Zhang

**Affiliations:** 1 State Key Laboratory of Superlattices and Microstructures, Institute of Semiconductors, Chinese Academy of Sciences, Beijing 100083, China; 2 College of Engineering, Bohai University, Jinzhou 121013, China; 3 Department of Physics, Bohai University, Jinzhou 121000, China

## Abstract

Thermoelectric effect is studied in an Aharonov-Bohm interferometer with an embedded quantum dot (QD) in the Coulomb blockade regime. The electrical conductance, electron thermal conductance, thermopower, and thermoelectric figure-of-merit are calculated by using the Keldysh Green's function method. It is found that the figure-of-merit *ZT *of the QD ring may be quite high due to the Fano effect originated from the quantum interference effect. Moreover, the thermoelectric efficiency is sensitive to the magnitude of the dot-lead and inter-lead coupling strengthes. The effect of intradot Coulomb repulsion on *ZT *is significant in the weak-coupling regime, and then large *ZT *values can be obtained at rather high temperature.

## 1. Introduction

Thermoelectric phenomenon, which involves the conversion between thermal and electrical energies, has attracted much theoretical and experimental interests in recent years [1,2]. However, except for specialized applications such as laboratory equipment and space missions, thermoelectric energy conversion technology has yet not widely been used due to its low efficiency [1,2]. The thermoelectric efficiency is usually characterized by the dimensionless figure-of-merit *ZT *= *S*^2^*GT=*(*κ*_el _+ *κ*_ph_), where *T *is the temperature of the system, the thermopower (Seebeck coefficient) *S *measures how large a voltage can be induced in response to a given temperature gradient, the electrical conductance *G *measures how easily charges can flow through the system to generate voltage drop, the electron thermal conductance *κ_el _*and phonon thermal conductance *κ*_ph _measure how hard heat can transfer across the system to maintain a temperature gradient [2].

To increase the magnitude of *ZT*, large thermopower, high electrical conductance, and low thermal conductance are required. However, according to the Wiedemann-Franz law [1,2], these physical quantities are interdependent in conventional bulk thermoelectric materials (the ratio *κ*_el_/*GT *remains constant). Increasing in the electrical conductance usually leads to a corresponding increase in the thermal conductance, and is always accompanied by a decrease in the thermopower [3,4]. Currently, bulk materials with highest *ZT *values are Bi_2_Te_3 _alloys with Sb, Sn, and Pb, such as Bi_0.5_Sb_1.5_Te_3_, whose *ZT *value is about one at room temperature [5]. To find real use in commercial products, however, thermoelectric materials with *ZT >*3 are required [1,2]. Until now, many approaches have been proposed to enhance the thermoelectric efficiency, and one of which is to reduce the system dimensionality. Therefore, thermoelectric effect in zero-dimensional quantum dot (QD) [5-7] has been intensively studied in the Coulomb blockade [8-14] and Kondo [15,16] regimes. The considerably enhanced thermoelectric efficiency in such devices was attributed to the strong violation of Wiedemann-Franz law by the Coulomb interaction [10] and the significant reduction of *κ*_ph _due to the strong phonon scattering by the interfaces between the nanostructures [13]. Experimentally, it has been shown that the maximum *ZT *value of 2 can be reached in PbSeTe QDs [17].

To date, most previous studies focused on single dot with one or multiple energy levels coupled to normal metal or ferromagnetic leads. Recently, parallel and serially coupled double QDs [18,19] structures and triple QDs [13] systems have also been studied. Liu and Yang [19] have investigated the thermal properties of double QDs molecular junction, and found that the Fano effect can improve the thermoelectric efficiency. However, as a typical system to study the Fano effect, the thermoelectric properties of an Aharonov-Bohm (AB) interferometer with an embedded QD has seldom been investigated. Blanter et al. [20] have studied the AB-type oscillations of thermopower in a QD ring geometry, but they mainly considered the effect of geometric phase in the absence of the intradot Coulomb interaction. In addition, Kim and Hershfield [21,22] have also investigated the influence of the AB flux on thermopower in the Kondo regime. To the best of the authors' knowledge, the figure-of-merit in a single QD AB ring structure has never been investigated, which is the motivation of this article.

In this article, we study the thermoelectric effect in a single QD ring with both Coulomb interaction and magnetic flux at room temperature. The electrical conductance, thermal conductance, thermopower, and thermoelectric figure-of-merit as functions of the QD energy level are investigated in the linear-response regime, where the temperature and the bias voltage differences between the two leads all tend to zero. We found that, different from symmetric coupled double QDs AB ring, high *ZT *values can be obtained even without the help of magnetic field. All the thermoelectric quantities oscillate with magnetic field with a period of 2π. The *ZT *magnitude will be suppressed except for the magnetic flux values are at *ϕ *= *n *(*n *+ 1) π */*2 (*n *= 0, 1, 2 . . .). Moreover, the figure-of-merit can be enhanced obviously if we choose weak dot-lead and inter-lead coupling strengths as well as strong intradot Coulomb interaction. By optimizing a number of parameters, *ZT *can be much larger than 3 at room temperature.

## 2. Model and method

The system can be described by the following Hamiltonian [23]:

(1) $$\begin{array}{c} H=\underset{k,\alpha ,\sigma }{\sum }\varepsilon_{k\alpha }c_{k\alpha \sigma }^{†}c_{k\alpha \sigma }+\underset{\sigma }{\sum }\varepsilon_{d}d_{\sigma }^{†}d_{\sigma }+Ud_{↑}^{†}d_{↑}d_{↓}^{†}d_{↓}+\underset{\sigma }{\sum }(t_{Ld}c_{kL\sigma }^{†}d_{\sigma }+t_{Rd}c_{kR\sigma }^{†}d_{\sigma }\\ \;+t_{LR}e^{i\phi }c_{kL\sigma }^{†}c_{kR\sigma }+\text{H}.\text{c}\text{.}), \end{array}$$

where $c_{k\alpha \sigma }^{†}\left(c_{k\alpha \sigma }\right)$ is the creation (annihilation) operator of the electrons with momentum *k*, spin *σ*, and energy *ε_kα _*in the *α *(*α *= *L*, *R*) lead. $d_{\sigma }^{†}\left(d_{\sigma }\right)$ creates (annihilates) an electron in the QD with spin *σ *and energy *ε_d_*. *U *is the intradot electron-electron Coulomb repulsion energy. *t_αd _*and *t_LR _*describes the energy-independent dot-lead and lead-lead tunneling couplings, respectively. Finally, the magnetic flux Φ threading through the ring gives rise to a phase factor *ϕ *= 2*π *Φ*/*Φ_0 _in the tunneling coupling term *t_LR_*.

By using the standard nonequilibrium Green's function method, the charge and heat currents flowing from the left lead into the right one can be derived as [2,23-25]

(2) $$\left(\begin{array}{c} J\\ Q \end{array}\right)=\frac{2}{h}\int d\varepsilon \left(\begin{array}{c} e\\ \varepsilon -\mu_{L} \end{array}\right)\text{Re}\;\left[t_{Ld}G_{dL\sigma }^{<}\left(\varepsilon \right)+t_{LR}G_{RL\sigma }^{<}\left(\varepsilon \right)\right].$$

The lesser Green functions can be obtained straightforwardly by using the standard Keldysh equation

(3) $$G_{\sigma }^{<}\left(\varepsilon \right)=G_{\sigma }^{r}\left(\varepsilon \right)\;g_{\sigma }^{r\;-\;1}g_{\sigma }^{<}g_{\sigma }^{a\;-\;1}G_{\sigma }^{a}\left(\varepsilon \right)+G_{\sigma }^{r}\left(\varepsilon \right)\Sigma_{\sigma }^{<}G_{\sigma }^{a}\left(\varepsilon \right),$$

where the first and the second terms describe the elastic and the inelastic transport, respectively. For our present case, we only consider the elastic transport which preserves the quantum coherence, so we can simply take $\Sigma_{\sigma }^{<}=0$. In addition, $g_{\sigma }^{r\;-\;1}g_{\sigma }^{<}g_{\sigma }^{a\;-\;1}$ is diagonal, with the matrix element $g_{dd\sigma }^{r\;-\;1}g_{dd\sigma }^{<}g_{dd\sigma }^{a\;-\;1}=0$ and $g_{\alpha \alpha \sigma }^{r\;-\;1}g_{\alpha \alpha \sigma }^{<}g_{\alpha \alpha \sigma }^{a\;-\;1}=2i\;f_{\alpha }\left(\varepsilon \right)/)\left(\pi \rho \right)$, where *ρ *is the density of states in the leads and *f_α _*(*ε*) = {1 + exp [(*ε *- *μ_α_*)/*k_B_T*]}^-1 ^is the Fermi distribution function for lead *α *with chemical potential *μ_α _*and temperature *T *.

By means of the Dyson equation, the retarded Green's function $G_{\sigma }^{r}\left(\varepsilon \right)$ can be given by $G_{\sigma }^{r}\left(\varepsilon \right)=g_{\sigma }^{r}\left(\varepsilon \right)+g_{\sigma }^{r}\left(\varepsilon \right)\;\Sigma_{\sigma }^{r}G_{\sigma }^{r}\left(\varepsilon \right)$. The diagonal matrix $g_{\sigma }^{r}\left(\varepsilon \right)$ represents the bare Green's function for electrons in the leads and the QD, in which $g_{\alpha \alpha \sigma }^{r}=-i\pi \rho$ and $g_{dd\sigma }^{r}=\left(\varepsilon -\varepsilon_{d}-U+Un_{\bar{\sigma }}\right)/\left[\left(\varepsilon -\varepsilon_{d}\right)\;\left(\varepsilon -\varepsilon_{d}-U\right)\right]$ The intradot electron occupation number *n_σ _*can be solved R self-consistently from the equation $n_{\sigma }=-i\;\int \left(d\omega /2\pi \right)G_{dd\sigma }^{<}\left(\varepsilon \right)$. The retarded self-energy $\Sigma_{\sigma }^{r}$ in the Dyson equation is composed of the elements denoting the dot-lead and the inter-lead tunneling coupling strengths. In this article, all the above matrices are of order 3.

By substituting $G_{dL\sigma }^{<}\left(\varepsilon \right)$ and $G_{RL\sigma }^{<}\left(\varepsilon \right)$ into Equation (2), the charge and heat currents can easily be rearranged into the Landauer formula form

(4) $$\left(\begin{array}{c} J\\ Q \end{array}\right)=\frac{2}{h}\;\int d\varepsilon \left(\begin{array}{c} e\\ \varepsilon -\mu_{L} \end{array}\right)\tau \left(\varepsilon \right)\left[f_{L}\left(\varepsilon \right)-f_{R}\left(\varepsilon \right)\right],$$

in which the transmission coefficient for the symmetric coupling case (*t_Ld _*= *t_Rd _*= *t_d_*) is

(5) $$\tau \left(\varepsilon \right)=2{\left(g_{\alpha \alpha \sigma }^{r\;-\;1}\right)}^{2}\left[{\left(g_{dd\sigma }^{r\;-\;1}\right)}^{2}t_{LR}^{2}+2g_{dd\sigma }^{r\;-\;1}t_{d}^{2}t_{LR}\cos\;\phi +t_{d}^{4}\right]/\Omega \;\left(\varepsilon \right),$$

with $\Omega \;\left(\varepsilon \right)=-4g_{dd\sigma }^{r\;-\;1}t_{d}^{2}t_{LR}\cos\phi \left[{\left(g_{dd\sigma }^{r\;-\;1}\right)}^{2}+t_{LR}^{2}\right]-{\left(g_{dd\sigma }^{r\;-\;1}\right)}^{2}{\left[{\left(g_{\alpha \alpha \sigma }^{r\;-\;1}\right)}^{2}+t_{LR}^{2}\right]}^{2}-4t_{d}^{4}\left[{\left(g_{dd\sigma }^{r\;-\;1}\right)}^{2}+t_{LR}^{2}\cos^{2}\phi \right]$. As we are interested in the linear-response regime, the chemical potentials and the temperatures of the two leads are set to be *μ_L _*= *μ_R _*= *μ *and *T_L _*= *T_R _*= *T*. After expanding the Fermi-Dirac distribution function to the first-order in Δ*T *and Δ*V*, Equation (4) can be written as [2]

(6) $$\left(\begin{array}{c} J\\ Q \end{array}\right)=\left(\begin{array}{cc} \frac{2e^{2}}{h}K_{0} & -\frac{2e}{hT}K_{1}\\ -\frac{2e}{hT}K_{1} & \;\;\frac{2}{hT}K_{2} \end{array}\right)\;\left(\begin{array}{c} \Delta V\\ \Delta T \end{array}\right),$$

where *K_n _*(*μ*, *T*) = *∫ dε *(-∂ *f */∂*ε*) (*ε *- *μ*)*^n ^τ *(*ε*). Correspondingly, the electrical conductance *G*, thermopower *S*, and electron thermal conductance *κ_el _*are, respectively, given by *G *= 2*e*^2 ^*K*_0 _(*μ*, *T*)/*h*, *S *= - *K*_1 _(*μ*, *T*)/[*eT K*_0 _(*μ*, *T*) ], $\kappa_{el}=2\left[K_{2}\left(\mu ,T\right)-K_{1}^{2}\left(\mu ,T\right)/K_{0}\left(\mu ,T\right)\right]/\left(hT\right)$.

## 3. Results and discussion

In the following numerical calculations, we fix *μ_L _*= *μ_R _*= 0 and *T *= 300*K *throughout the article. The local density of states in the leads *ρ *is chosen as the energy unit and set to be 1. We also chose symmetric dot-lead coupling *t_Ld _*= *t_Rd _*= *t_d_*.

**Figure 1:**
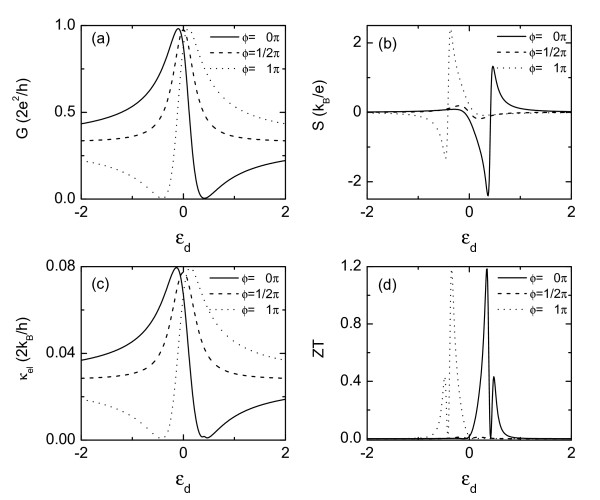
**Electrical conductance *G *(a), thermopower ***S ***(b), electron thermal conductance *κ_el _*(c), and thermoelectric figure of merit *ZT *(d) versus the intradot level ***ε_d _***for *ϕ *= 0, *π/*2, and *π*, respectively**. Other parameters are *t_d _*= 0.2, *t_LR _*= 0.1, and *U *= 0.

First, we discuss the results with different magnetic flux in the absence of Coulomb interaction and phonon thermal conductance. As shown in Figure 1a, the electrical conductance is composed of a Fano peak at the bonding state at $\varepsilon_{d}=2\;\cos\;\phi t_{d}^{2}t_{LR}/\left(t_{LR}^{2}-\pi^{-2}\right)$, and a Fano dip at antibonding state $\varepsilon_{d}=\sec\phi t_{d}^{2}\left(\cos2\phi t_{LR}^{2}+\pi^{-2}\right)/t_{LR}\left(t_{LR}^{2}+\pi^{-2}\right)$[19,26]. The Fano lineshape arises from the quantum interference effect when electrons are propagating through the AB ring via two paths: one is directly through the continuum level of the bridge between the two leads, and the other is through the resonance level of the QD. Now constructive and destructive interferences, respectively, correspond to resonant enhancement and resonant suppression of the transmission [26]. When there is no magnetic flux (i.e., *ϕ *= 0) as indicted by the solid line, the electrical conductance show a typical Fano resonance. Increasing the magnetic flux *ϕ *from 0, decoherence effect is increased and the asymmetric Fano line shape gradually evolves into a Lorentzian one at *ϕ *= *π/*2 (dotted curve). Further increasing *ϕ*, the Fano resonance emerges again, but with an opposite Fano tail as compared to the case of 0 *< ϕ < π/*2.

As shown in Figure 1b, the thermopower is enhanced by the Fano effect and changes sign around the antiresonance state. It varies rather sharply and has two maximums on each side of the Fano dip. Different from the case of *ϕ *= (*n *+ 1)*π/*2, the magnitudes of the two maximums are different from each other due to the asymmetry of the Fano resonance in the transmission spectra. The thermopower approaches to be zero at exactly the Fano antiresonance state due to electron-hole symmetry. At that state, the voltage drop induced by the electrons are cancelled by that of the holes, which are flowing in the opposite direction to the electrons. The electrons above (below) the antiresonance state in the left lead are less (more) than those in the right lead because of the temperature gradient that induces the thermoelectric phenomenon, resulting in the sign change of the thermopower. Owing to the fact that both the conductance *G *and the electronic thermal conductance *·κ*_el _are approximately proportional to the transmission *τ_σ _*(*μ*), the electronic thermal conductance resembles the electric conductance as shown in Figure 1c. One distinction between therm, however, is worth mentioning. The thermal conductance develops a small peak at the energy of Fano antiresonance state, which can be interpreted as follows: when the system is in the antiresonance state, current associated with electrons is compensated by current associated with holes tunneling in the opposite directions, leading to a zero conductance. But, the heat transferred by electrons cannot be compensated by the holes because of the energy difference between them. Thus, the thermal conductances from the electrons and the holes add constructively, giving rise to a peak in the electron thermal conductance. Once the behaviors of *G*, *S*, and *κ*_el _are known, the properties of the figure-of-merit *ZT *can be understood accordingly. *ZT *exhibits two peaks and a valley as shown in Figure 1d. These two peaks correspond to the maximums of the thermopower, and the valley is at the energy state of *S *= 0. It is worth noting that the *ZT *value is very small for *ϕ *= (*n*+1) *π/*2 because of the disappearance of the Fano effect. Moreover, it can easily be seen from Equation (5) that *G*, *S*, *κ*_el_, and *ZT *are all functions of cos *ϕ*.

**Figure 2:**
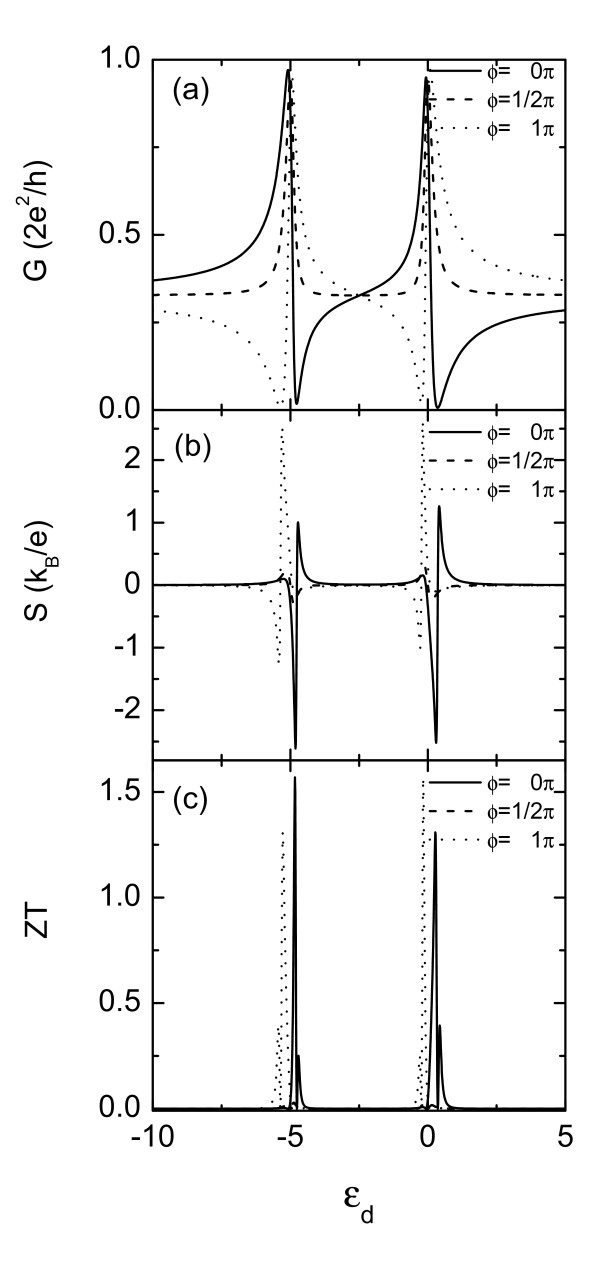
**Electrical conductance *G *(a), thermopower ***S ***(b), and thermoelectric figure of merit *ZT *(c) versus the intradot level *ε_d _*for *U *= 5**. The other parameters are the same as those of Figure 1.

We next study the influence of the intradot Coulomb interaction on the relevant thermoelectric quantities. Figure 2a depicts the conductance as a function of the dot level with *U *= 5*: *The conductance curves are dominated by two Fano peaks (dips) spaced by the Coulomb interaction energy *U*. Owing to the electrical conductance, *G *= (2*e*^2 ^*/h*) *K*_0 _is weighted by the derivative of the Fermi function, the Fano peak (dip) around the energy level of *ε_d _*= *-U *is slightly larger than that at *ε_d _*= 0 at finite temperature. Similar to the case of *U *= 0, the thermopower is enhanced obviously at the two sides of the antiresonance dips (see Figure 2b). The magnitudes of the maximums of the thermopower at *ε_d _*= 0 are different from those at *-U*, which can be attributed to the behavior of the electric conductance in Figure 2a. Due to the property of the thermopower, the *ZT *value is significantly enhanced with the help of *U*, which is shown in Figure 2c. The maximum of *ZT *can approach 1.55 which is greater than that of the *U *= 0 case.

**Figure 3:**
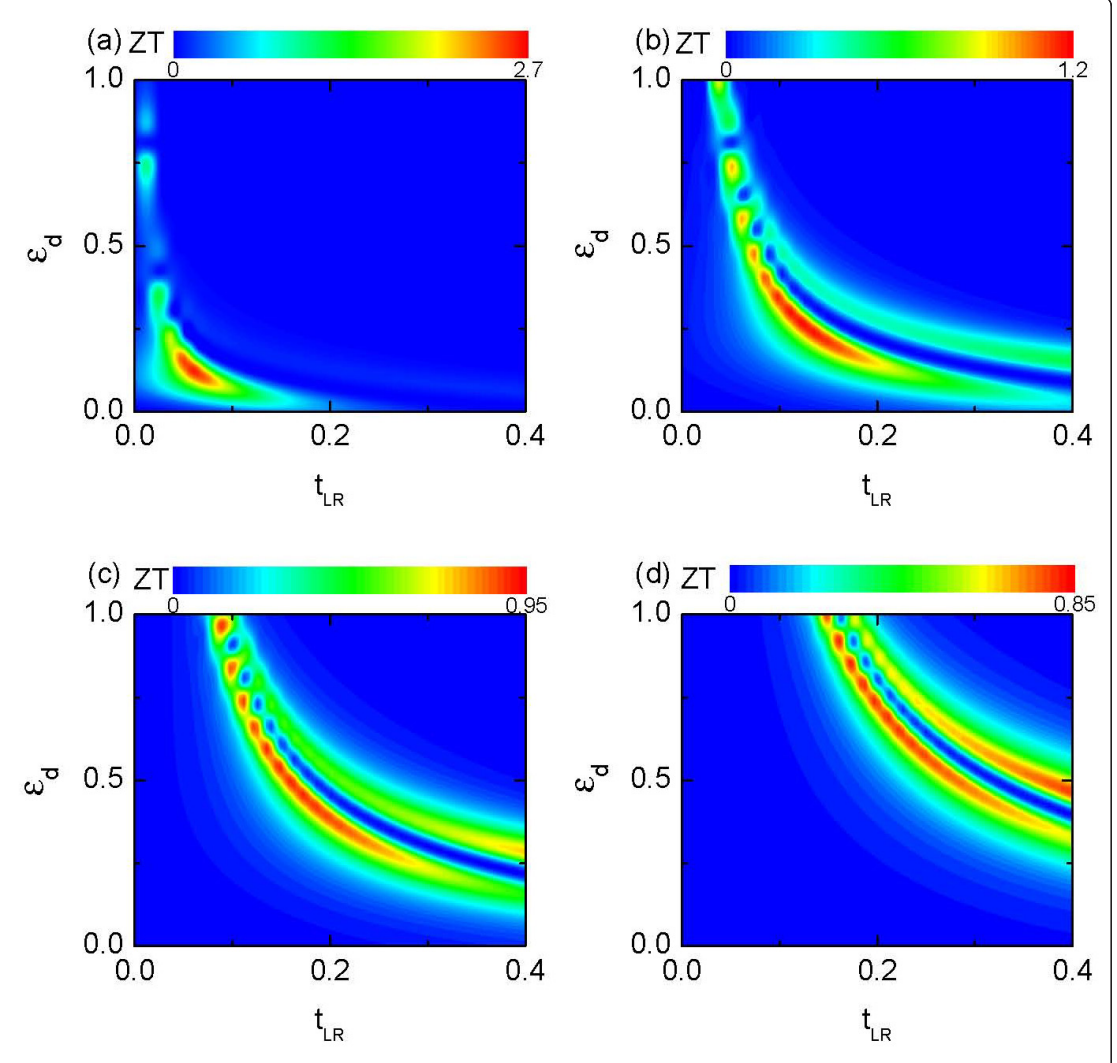
**Figure-of-merit *ZT *as a function of the intradot level *ε_d _*and inter-lead coupling strengths ***t_LR _***for different dot-lead coupling strengths: (a) *t_d _*= 0.1; (b) *t_d _*= 0.2; (c) *t_d _*= 0.3; (d) *t_d _*= 0.4**. The magnetic flux and the Coulomb interaction are all set to be zero.

Figure 3 depicts the figure-of-merit *ZT *for different intradot level *ε_d _*and the bridge coupling strength *t_LR _*with *U *= 0. Panels (a)-(d) refer to the cases of different values of dot-lead coupling strengths of *t_d _*= 0.1, 0.2, 0.3, and 0.4, respectively. The figure-of-merit *ZT *is found to be prominent around $\varepsilon_{d}=t_{d}^{2}/t_{LR}$ and *t_LR _*= *t_d _/*2. The weaker the coupling between the dot and the leads is, the larger the *ZT *value is. This result is consistent with previous studies [27], where it was shown that weak coupling strength can lead to strong violation of the Wiedemann-Franz law, resulting in a large *ZT *value. Here, we do not consider such extremely weak coupling case, although tremendous large *ZT *is expected to take place. This is because in the linear response region one assumes that the temperature difference is the smallest energy scale. If we set *t_d _*→ 0, the coupling strength becomes comparable to such a scale, and the linear response approximation may break down and leads to unreasonable results.

**Figure 4:**
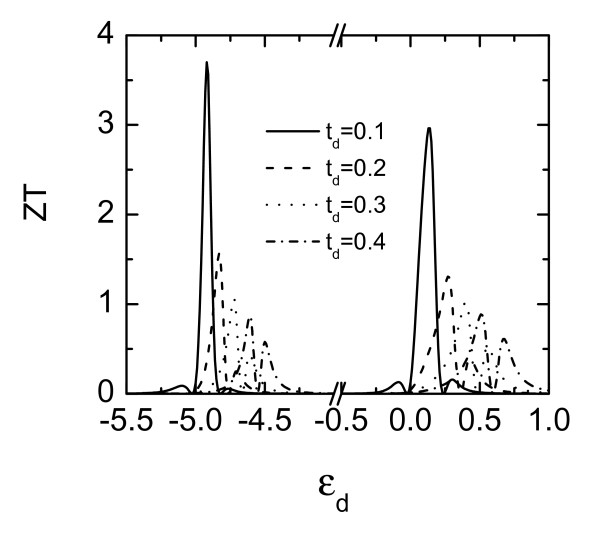
**Figure-of-merit versus the intradot level *ε_d _*for *t_d _*= 0.1, 0.2, 0.3, 0.4**. Other parameters are *t_LR _*= *t_d _/*2, *U *= 5, and *ϕ *= 0.

Next, we investigate the impact of the Coulomb interaction on *ZT *for various dot-lead coupling strengths. As shown in Figure 4, the Coulomb interaction has a pronounced impact on the thermoelectric efficiency as the coupling strength is decreased. This is because the transmission coefficient is proportional to the dot-lead and the inter-lead tunneling strengths. When the QD is weakly coupled to external leads, electrons are difficult to tunnel between the QD and the leads. The thermopower (or the electrical bias) induced by the temperature gradient will be enhanced, leading to higher *ZT *value. Finally, we briefly discuss the influence of phonon thermal conductance *κ*_ph _on the figure-of-merit. It has been proved that similar to the electrical conductance, thermal transport in mesoscopic phonon systems also has a quantized unit $\kappa_{\text{ph}}=\pi^{2}k_{B}^{2}T/\left(3h\right)$ at low temperature [1,2]. For higher temperature, electron-phonon interaction influences both the dot level position and dot-lead coupling strength [28,29] and has negative contribution to the magnitude of the figure-of-merit as is seen from its definition. Nevertheless, the Fano effect is robust against the presence of phonon [30], and we expect the enhanced thermoelectric efficiency will hold qualitatively true with such an interaction but with shifted dot levels' position and weakened magnitude.

## 4. Conclusion

We have studied the thermoelectric properties in an AB ring with an embedded QD in the Coulomb blockade regime. Our results show that the *ZT *value can be very high due to the Fano effect, which arises from the quantum interference phenomenon. Around the Fano dip, the transmission coefficient suffers an abrupt change leading to an increase in the thermopower. This fact and the very small electric thermal conductance around the antiresonance lead to a large thermoelectric efficiency. Moreover, the coupling strengths between the leads and the intradot Coulomb interaction exert significant impacts on the figure-of-merit. The influence of the Coulomb interaction is more pronounced in the weak coupling case. The *ZT *value can reach 3 or larger at room temperature for some selected parameters.

## Competing interests

The authors declare that they have no competing interests.

## Authors' contributions

JZ and FC established the theoretical formalism. JZ, XDL, and KC Zhang carried out numerical calculations and the establishment of the figures. FC conceived of the study, and participated in its design and coordination. All the authors read and approved the final manuscript.
